# Effects of sodium–glucose cotransporter 2 inhibitors in patients with cancer and diabetes mellitus: a systematic review and meta-analysis

**DOI:** 10.1093/ehjcvp/pvaf028

**Published:** 2025-04-25

**Authors:** Giuseppina Novo, Cristina Madaudo, Antonio Cannatà, Pietro Ameri, Daniela Di Lisi, Daniel I Bromage, Alfredo Ruggero Galassi, Giorgio Minotti, Alexander R Lyon

**Affiliations:** Cardiology Unit, University Hospital ‘Paolo Giaccone’, Via del vespro 129, 90127 Palermo, Italy; Department of Health Promotion, Mother and Child Care, Internal Medicine and Medical Specialties (ProMISE) University of Palermo, Piazza delle Cliniche 2, 90127 Palermo, Italy; Department of Health Promotion, Mother and Child Care, Internal Medicine and Medical Specialties (ProMISE) University of Palermo, Piazza delle Cliniche 2, 90127 Palermo, Italy; Department of Cardiovascular Sciences, British Heart Foundation Centre of Research Excellence, School of Cardiovascular Medicine, Faculty of Life Science, King’s College London, The James Black Centre, 125 Coldharbour Lane, London SE5 9NU, UK; Department of Cardiovascular Sciences, British Heart Foundation Centre of Research Excellence, School of Cardiovascular Medicine, Faculty of Life Science, King’s College London, The James Black Centre, 125 Coldharbour Lane, London SE5 9NU, UK; Department of Internal Medicine, University of Genova, Viale Benedetto XV, 6, 16132 Genoa, Italy; IRCCS Ospedale Policlinico San Martino, Largo Rosanna Benzi, 10, 16132 Genoa, Italy; Cardiology Unit, University Hospital ‘Paolo Giaccone’, Via del vespro 129, 90127 Palermo, Italy; Department of Cardiovascular Sciences, British Heart Foundation Centre of Research Excellence, School of Cardiovascular Medicine, Faculty of Life Science, King’s College London, The James Black Centre, 125 Coldharbour Lane, London SE5 9NU, UK; Cardiology Unit, University Hospital ‘Paolo Giaccone’, Via del vespro 129, 90127 Palermo, Italy; Department of Health Promotion, Mother and Child Care, Internal Medicine and Medical Specialties (ProMISE) University of Palermo, Piazza delle Cliniche 2, 90127 Palermo, Italy; Department of Medicine and Unit of Clinical Pharmacology, University and Fondazione Policlinico Universitario Campus Bio-Medico, Via Álvaro del Portillo 200, 00128 Rome, Italy; Cardio-Oncology Centre of Excellence, Royal Brompton Hospital, Fulham Wing, Dovehouse Street, London SW3 6JY, UK

**Keywords:** Cardio-oncology, Cardiotoxicity, SGLT2 inhibitors (SGLT2i), Cancer therapy-related cardiac dysfunction (CTRCD), Heart failure hospitalization, Preventive cardiology

## Abstract

**Aims:**

Cardiovascular disease and cancer represent significant global health challenges. An overlap between oncology and cardiology is compounded by cancer therapies, which are known to have cardiotoxic effects. Sodium–glucose cotransporter 2 inhibitors (SGLT2i), initially developed for treating diabetes, have shown promising cardiovascular benefits in non-cancer populations, particularly in preventing heart failure (HF) and reducing HF-related hospitalization and mortality in large randomized controlled trials (RCTs) across the spectrum of left ventricular ejection fraction. However, their potential cardioprotective role in cancer patients remains unclear. This systematic review and meta-analysis evaluated cardiovascular outcomes in cancer patients with type 2 diabetes undergoing chemotherapy with concomitant use of SGLT2i compared with those not using SGLT2i. Subgroup analyses were performed to explore patients without baseline HF and patients treated exclusively with anthracyclines.

**Methods and results:**

A systematic review identified 11 observational retrospective studies (*n* = 104 327 patients). Based on the National Institutes of Health Quality Assessment Tool checklist, two studies were at moderate risk of bias, while all other included studies had a low risk of bias. Meta-analysis indicated that the use of SGLT2i was associated with a significant reduction in all-cause mortality [0.47, 95% confidence interval (CI) 0.33–0.67, *P* < 0.0001] and risk of HF hospitalization (0.44, 95% CI 0.27–0.72, *P* = 0.001).

**Conclusion:**

The use of SGLT2i may be associated with a significant reduction in all-cause mortality and risk of HF hospitalization in actively treated cancer patients with Type 2 diabetes. Our study highlights the need for further investigation through prospective RCTs to confirm the efficacy and safety of SGLT2i in attenuating cardiotoxicity and supporting cardiovascular health in oncology settings.

## Graphical Abstract

**Graphical Abstract:**
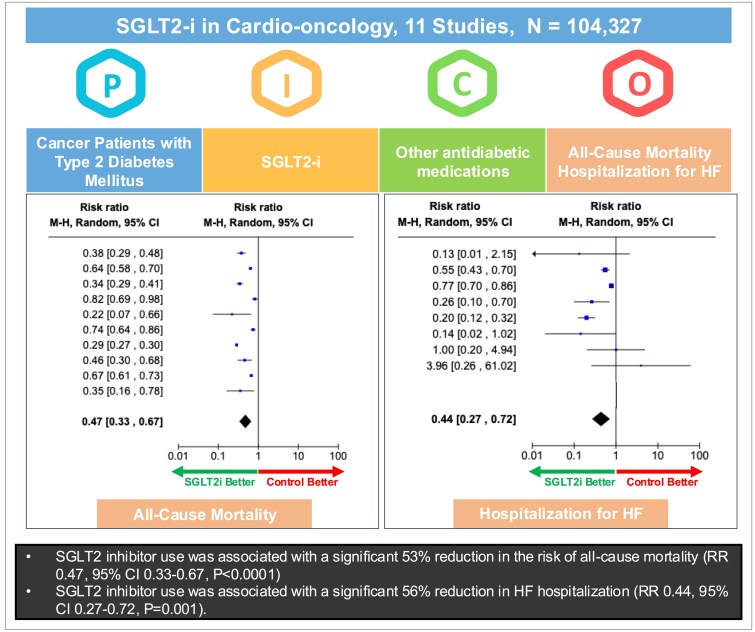
This summarizes 11 studies involving 104,327 participants selected based on the Population, Intervention, Comparison, and Outcomes strategy. These studies evaluated the benefits of SGLT2i compared with non-users, focusing on cancer patients with diabetes mellitus. The outcomes highlight significant reductions in all-cause mortality and HF hospitalizations with SGLT2i use. 95% CI, 95% confidence interval; HF, heart failure; RR, risk ratio; SGLT2i, sodium–glucose cotransporter 2 inhibitors.

## Introduction

Cardiovascular (CV) disease (CVD) and cancer are among the two leading causes of mortality and morbidity worldwide, particularly in Western countries, with millions of cancer survivors being at an increased risk of developing CVD. In the USA alone, it is estimated that there are over 17 million cancer survivors who may be at risk of CVD, with a continuously growing trend that is expected to rise further in the coming years.^1^

Many cancer patients experience cancer therapy-related cardiac dysfunction (CTRCD), particularly heart failure (HF), which adds complexity to managing their long-term health.^2,3^ The European Society of Cardiology guidelines divide CTRCD into asymptomatic and symptomatic, related to the occurrence of typical symptoms of HF up to severe forms requiring hospitalization.^4^ Early detection of CTRCD has been shown to reduce the burden of cardiac dysfunction.^5,6^ Therefore, the echocardiography assessment of left ventricular ejection fraction (LVEF) and global longitudinal strain (GLS) plays a crucial role.^7,8^ Furthermore, emerging parameters such as global atrioventricular strain and myocardial work indices have further improved the ability to detect cardiotoxicity at earlier stages.^9–11^ Early diagnosis and prompt initiation of neurohormonal therapy are recommended for cardioprotection and LVEF recovery.^5,6^

Sodium–glucose cotransporter 2 inhibitors (SGLT2i), a class of oral diabetes drugs that increase urinary glucose excretion by blocking kidney reabsorption, have been effective in reducing CV death and hospitalizations for HF (HHF) in both diabetic and non-diabetic patients^12,13^ and also in HF patients with different LVEF.^14,15^ Certainties are limited in the field of cardio-oncology. In a prospective case–control study enrolling 76 patients with breast cancer at high risk of CTRCD on anthracycline therapy without a previous diagnosis of HF, empagliflozin use was associated with a reduction in CTRCD at 6-month follow-up compared with the control group (6.5% vs. 35.5%, *P* 0.005).^16^ To date, in this specific setting of patients undergoing chemotherapy, there are no other randomized controlled trials (RCTs) that evaluate harder outcomes such as CV death or HHF.

Other observational studies have indicated potential CV benefits of SGLT2i in cancer patients.^17,18^ Despite these results, the specific role of SGLT2i in preventing CTRCD remains uncertain.^19^ We performed a systematic review and meta-analysis of available evidence to assess the utility of SGLT2i on all-cause mortality and HHF in cancer patients with Type 2 diabetes mellitus undergoing chemotherapy.

## Methods

### Protocol and search strategy

This systematic review was conducted according to the Preferred Reporting Items for Systematic Reviews and Meta-Analysis (PRISMA) statement.^20^ According to the PRISMA, a Population, Intervention, Comparison, and Outcomes strategy was performed (*Table 1*).

**Table 1:** PICOS (Population, Intervention, Comparison, Outcomes, and Study) data for formulating eligibility criteria in the meta-analysis

Patient group	Cancer patients with diabetes mellitus
Intervention	SGLT2i
Comparator	Other antidiabetic medications
Outcome	All-cause mortality Hospitalization for heart failure
Setting	All study designs

The focus was on studies evaluating all cancer patients undergoing all possible antitumour therapies with Type 2 diabetes mellitus treated with SGLT2i compared with other antidiabetic medications not including the use of SGLT2i. Only studies with at least 12 months of follow-up were considered since acute CV events are usually rare in this patient setting. Observational studies with primary or secondary CV outcomes (hospitalization for HF and CV mortality) or all-cause mortality were selected and included. All types of studies were considered. No RCTs were available, except for one with a short 6-month follow-up that evaluated the incidence of subclinical dysfunction (defined as decrease in LVEF of at least 10% from baseline to a final LVEF of <50% and/or a relative decrease of at least 15% in GLS from baseline) in a high-risk breast cancer population.^16^ However, due to its limited follow-up and the definition of CTRCD based solely on subclinical dysfunction detected by echocardiographic assessment, we could not include it in our analysis. Articles not in English were excluded to ensure accessibility and consistent understanding of the data, avoiding potential translation errors. Duplicates, review articles, unpublished data, ongoing studies, non-peer-reviewed articles, and abstracts were excluded. Studies not involving humans were also excluded to guarantee the findings’ clinical relevance and direct applicability.

A researcher (C.M.) systematically searched PubMed, OVID, Medline, Embase, and the Cochrane Library for publications prior to 1 November 2024 using predefined keywords and Medical Subject Headings, including ‘SGLT2 inhibitors’, ‘SGLT2i’, ‘sodium–glucose transporter 2 inhibitors’, ‘cardiotoxicity’, ‘cardiotoxic’, ‘neoplasm’, ‘cancer’, ‘chemotherapy’, and their synonyms to obtain relevant literature (see Supplementary material online, *Table S1*). Furthermore, potential candidate papers were manually checked in the references of the included studies. Two researchers independently (G.N. and C.M.) examined the eligibility and screened the titles and abstracts of all identified potential studies (Level 1 screening). Then, the two researchers continued with a full-text review to finalize the study selection (Level 2 screening). Discrepancies were discussed and resolved by consensus with a third reviewer (A.C.). Extracted data included the first author’s name, year of publication, country, study design, baseline demographic and clinical characteristics of the population, gender, exposed group, observation group, cancer type, therapies, outcomes, and average follow-up years. This study does not require ethical approval or patient consent. The research protocol was registered on PROSPERO (ID: CRD42025625967).

### Outcomes and subgroup analyses

The primary outcome of the study was to evaluate the association between SGLT2i and all-cause mortality and HHF in cancer patients with Type 2 diabetes undergoing antineoplastic treatments. Subgroup analyses were performed to explore two specific populations: patients without baseline HF, with the aim of assessing the potential preventive role of SGLT2i in preserving cardiac function, and patients treated exclusively with anthracyclines. The focus on anthracyclines was chosen because their cardiotoxicity, particularly the risk of HF, is well established in patients receiving these treatments. Evaluating the effects of SGLT2i in this subgroup is crucial to determine their potential role in mitigating such cardiotoxic outcomes.

### Quality assessment

Two researchers (G.N. and C.M.) independently evaluated the quality of included studies using the National Institutes of Health Quality Assessment Tool (NIH-QAT), including 12 items, and any inconsistencies or disputes were settled by a third reviewer (A.C.). Each study was assessed as low risk of bias (9–10 criteria met), moderate risk of bias (7–8 criteria met), or high risk of bias (<5 criteria met).

### Sensitivity analysis

We also conducted a sensitivity analysis that excluded studies in which the chemotherapy regimen was not specified,^21–23^ the study with the shortest follow-up (1 year),^24^ and one study in which the cohort of patients receiving SGLT2i was older, had a significantly higher prevalence of hypertension, diabetes mellitus, dyslipidaemia, ischaemic heart disease, chronic kidney disease, end-stage renal disease, and elevated glycated haemoglobin, and were predisposed to greater susceptibility to complications, leading to higher rates of both hospitalization and mortality (not statistically significant compared with the control population).^25^ Additionally, one study was excluded because it considered death from any cause as a competing risk of HHF and not as an outcome; therefore, it could not be included in the sensitivity analysis for all-cause mortality and was also excluded from the analysis of HHF.^26^

### Statistical analysis

The effects of SGLT2i on each outcome were evaluated using the Mantel–Haenszel method, which was used to calculate the pooled risk ratio (RR) with a 95% confidence interval (CI). Anticipating heterogeneous results, a random-effects model was chosen. Heterogeneity among studies was assessed using χ^2^ and Higgins *I*² index, with thresholds of 30%, 50%, and 75% corresponding to low, moderate, and high heterogeneity.^27^ To manage heterogeneity, both subgroup and sensitivity analyses were performed. Subgroup analyses were stratified by the absence of HF at baseline and anthracycline use. Funnel plots were used to examine potential publication bias. A two-tailed level of significance was denoted as *P* < 0.05. All statistical analyses were performed using the Review Manager software (version 5.4, The Cochrane Collaboration, 2020).

## Results

### Study selection

**Figure 1:**
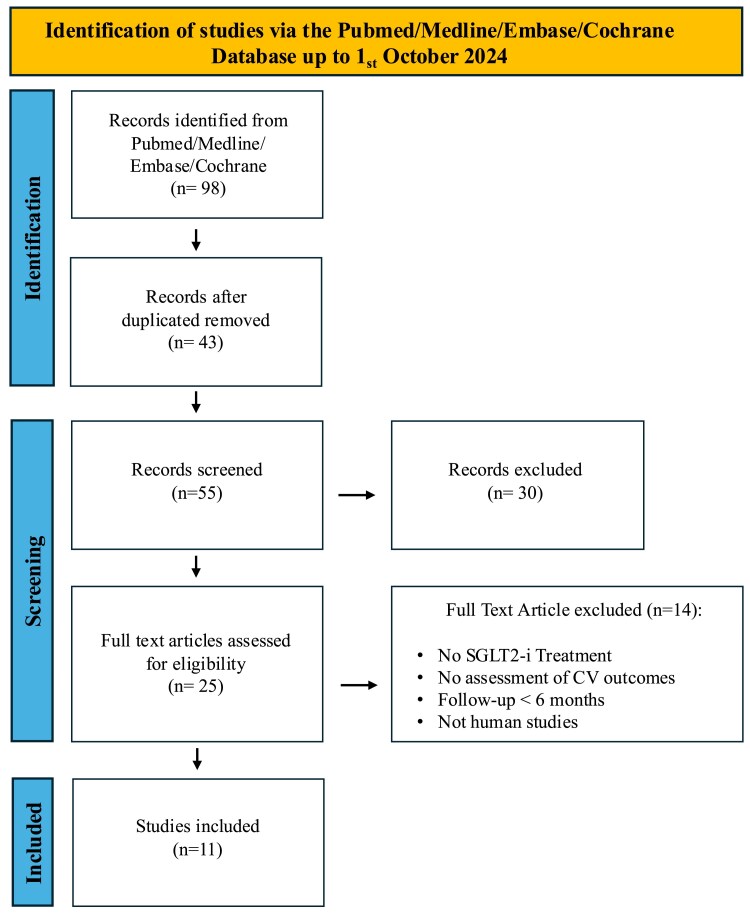
Flowchart of the selection process for the included studies.

The initial literature search identified 98 articles (*Figure 1*). After removing 43 duplicates and screening titles and abstracts, 25 full-text articles were assessed for eligibility. Fourteen articles were excluded because they were not studies on SGLT2i or had no CV outcomes, were not studies in humans, or had a reported follow-up of <12 months. Given the lack of RCTs on this topic, the focus was on prospective or retrospective observational studies that assessed the impact of SGLT2i on CV outcomes in cancer patients undergoing chemotherapy. Eleven studies involving a total of 104 327 patients were included in the review and quantitative meta-analysis. *Table 2* summarizes the characteristics and the primary endpoints of the included studies. Full details of the included studies are provided in Supplementary material online, *Table S2*.

**Table 2:** Baseline characteristics of the selected studies

First author and years of publication	Population			Country	LVEF (%)	Cancers	Chemotherapy	Primary outcome	Follow-up (years)
	Overall	SGLT2i	No SGLT2i						
Gongora (2022)^28^	128	32	96	USA	63	Breast (24%) Other cancers (76%)	Anthracyclines (100%)	HF incidence, HF admissions, development of cardiomyopathy or arrhythmias	1.5
Chiang (2022)^29^	1756	878	878	Taiwan	NS	Breast (11%) Other cancers (89%)	Anthracyclines (8%) Others (92%)	HF hospitalization and all-cause Mortality	1.6
Hendrix (2022)^21^	3185	137	3048	USA	NS	Hepatocellular carcinoma (100%)	NS	All-cause mortality	1.8
Abdel-Qadir (2023)^26^	933	99	834	Canada	NS	Breast (34.5%) Other cancers (65.5%)	Anthracyclines (100%)	newly incident HF, HF admissions, arrhythmias, or a > 10% absolute decline in LVEF	1.6
Huang (2023)^23^	50 133	16 711	33 422	Taiwan	NS	Breast (20.4%) Other cancers (79.6%)	NS	All-cause mortality	4.5-4.8
Luo (2023)^22^	24 915	531	24 384	USA	NS	Non-small cell lung cancer (100%)	NS	All-cause mortality	1.3
Hwang (2023)^30^	3116	779	2337	South Korea	NS	Breast (49%) Other cancers (51%)	Anthracyclines (100%)	HF hospitalization, AMI, ischaemic stroke, all-cause mortality	3.4
Avula (2024)^31^	1280	640	640	USA	20	Breast (15.5%) Other cancers (84.5%)	Anthracyclines (20%) Others (80%)	HF exacerbations and all-cause mortality	2
Fath (2024)^25^	1412	706	706	USA	63	Breast (25.7%) Other cancers (74.3%)	Anthracyclines (100%)	New- onset HF	2
Perelman (2024)^32^	119	24	95	Israel	53.5	Breast (5%) Other cancers (95%)	ICIs (100%)	All-cause mortality	2.4
Bhatti (2024)^24^	17 350	8675	8675	USA	NS	Breast (26%) Other cancers (74%)	Anthracyclines (25%) Others (75%)	CTRCD	1

LVEF, left ventricular ejection fraction; NS, not specified; SGLT2i: sodium–glucose cotransporter 2 inhibitors

### Study characteristics

Seven studies were conducted in North America (USA and Canada),^21,22,24–26,28,31^ three in Asia (Taiwan and South Korea),^23,29,30^ and one in Israel.^32^ Studies periods ranged from January 2010 to August 2022. The total number of patients in the SGLT2i group was 29 212 compared with 75 115 in the control group. The average age of patients ranged from 56 to 77 years. Where reported, chemotherapy regimens included anthracycline chemotherapy, anti-HER2 therapies, alkylating agents, antimetabolites, platinum-based chemotherapy, tyrosine kinase inhibitors, immune checkpoint inhibitors (ICIs), vascular endothelial growth factor (VEGF) inhibitors, epidermal growth factor receptor (EGFR) antagonists, antimicrotubule agents, aromatase inhibitors, proteasome inhibitors, and radiotherapy. Chemotherapy type was not specified in three studies^21–23^(*Table 2*). Four studies included only patients receiving anthracycline chemotherapy.^25,26,28,30^ Eight of these studies included multiple cancer types, while two studies focused on a single tumour type, hepatocellular carcinoma, or non-small cell lung cancer, respectively.^21,22^ Average follow-up durations ranged from 1 to 4.8 years. In the selected studies, the incidence of adverse events was similar between the two study groups (see Supplementary material online, *Table S2*). Genitourinary infection is the most common adverse event followed by hypoglycaemia especially if in combination with metformin.

### Results of the quality assessment

**Figure 2:**
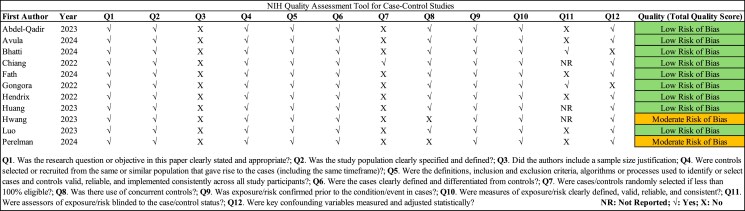
Quality assessment of the included studies using the NIH-QAT.

Based on the NIH-QAT checklist, nine studies had a low risk of bias and two studies had a moderate risk of bias (*Figure 2*). The least frequently reported item in the included studies was ‘Item 11’. This is likely because the included studies were observational rather than RCTs, where blinding of assessors is typically not specified or feasible. In observational studies, exposure and risk data are often collected retrospectively or during routine clinical practice, making blinding less applicable and more challenging to implement. Further information about the risk of bias assessment is described in *Figure 2*.

### Association with outcomes

#### All-cause mortality

**Figure 3:**
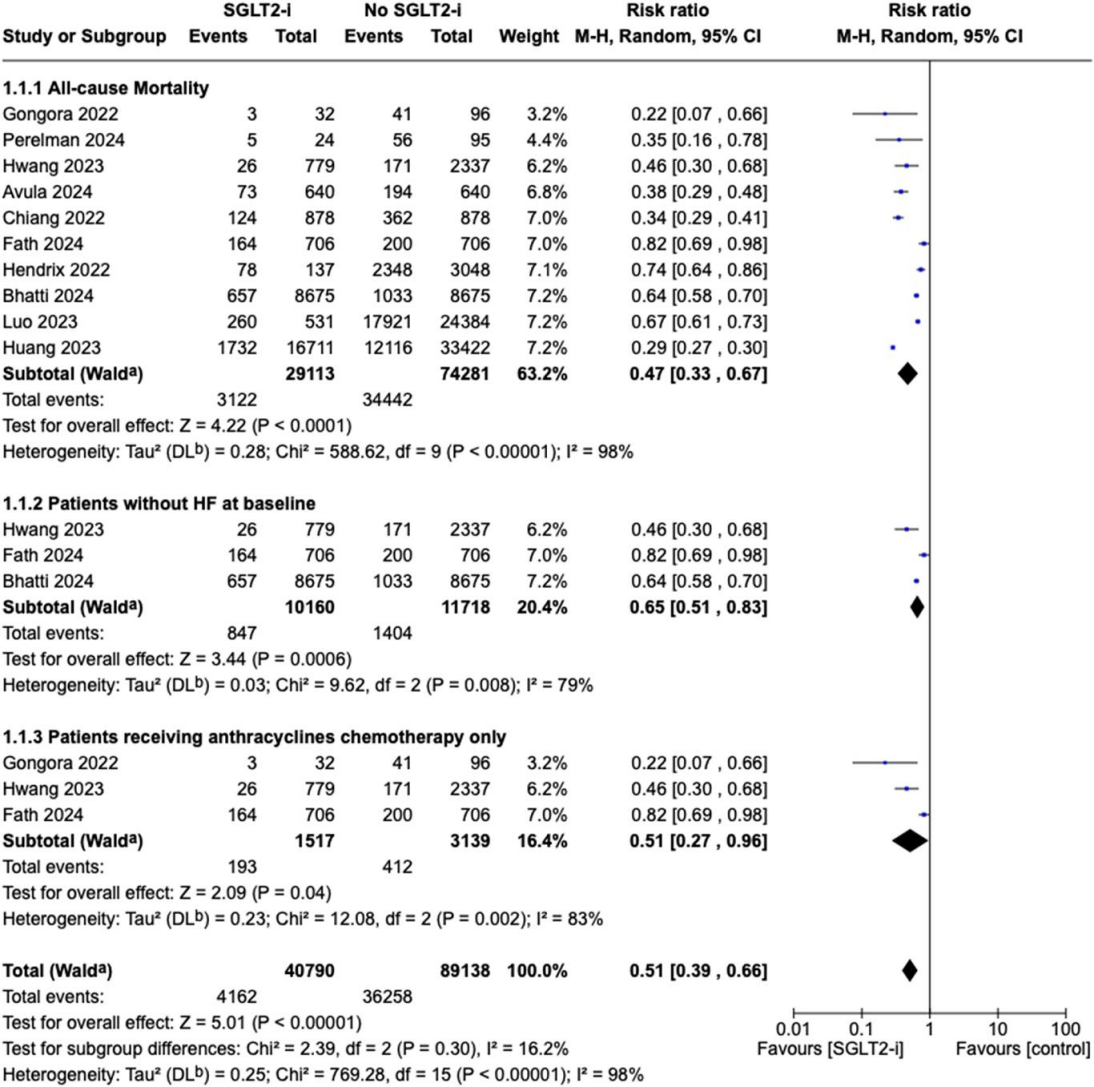
Forest plots of all-cause mortality. (*A*) All included studies. (*B*) Subgroup analysis of all-cause mortality in patients without heart failure at baseline. (*C*) Subgroup analysis of patients receiving anthracyclines chemotherapy only.

Ten studies included in the quantitative meta-analysis reported all-cause mortality as an outcome. One study was excluded because it considered death from any cause as a competing risk of HF hospitalization and not as an outcome.^26^ As shown in *Figure 3*, SGLT2i use was associated with a significantly lower mortality risk than non-use (RR 0.47, 95% CI 0.33–0.67, *P* < 0.0001). However, high heterogeneity between studies was found in this pooled result (*I*^2^ = 98%).

Subgroup analysis limited to three studies that excluded patients with HF at baseline found that the association with lower mortality remained significant (RR 0.65, 95% CI 0.51–0.83, *P* = 0.0006), despite high heterogeneity (*I*^2^ = 79%) (*Figure 3*). Subgroup analysis restricted to studies with only anthracycline chemotherapy showed a significantly lower risk of all-cause mortality in patients who used SGLT2-i (RR 0.51, 95% CI 0.27–0.96, *P* = 0.04; *I*^2^ = 83%) (*Figure 3*).

The sensitivity analysis, including five of the 10 selected studies, confirmed that SGLT2i use was associated with a significantly lower mortality risk than non-use (RR 0.36, 95% CI 0.32–0.41, *P* < 0.00001) with no heterogeneity (*I*^2^ = 0%) (see Supplementary material online, *Figure S1*).

#### Heart failure hospitalization

**Figure 4:**
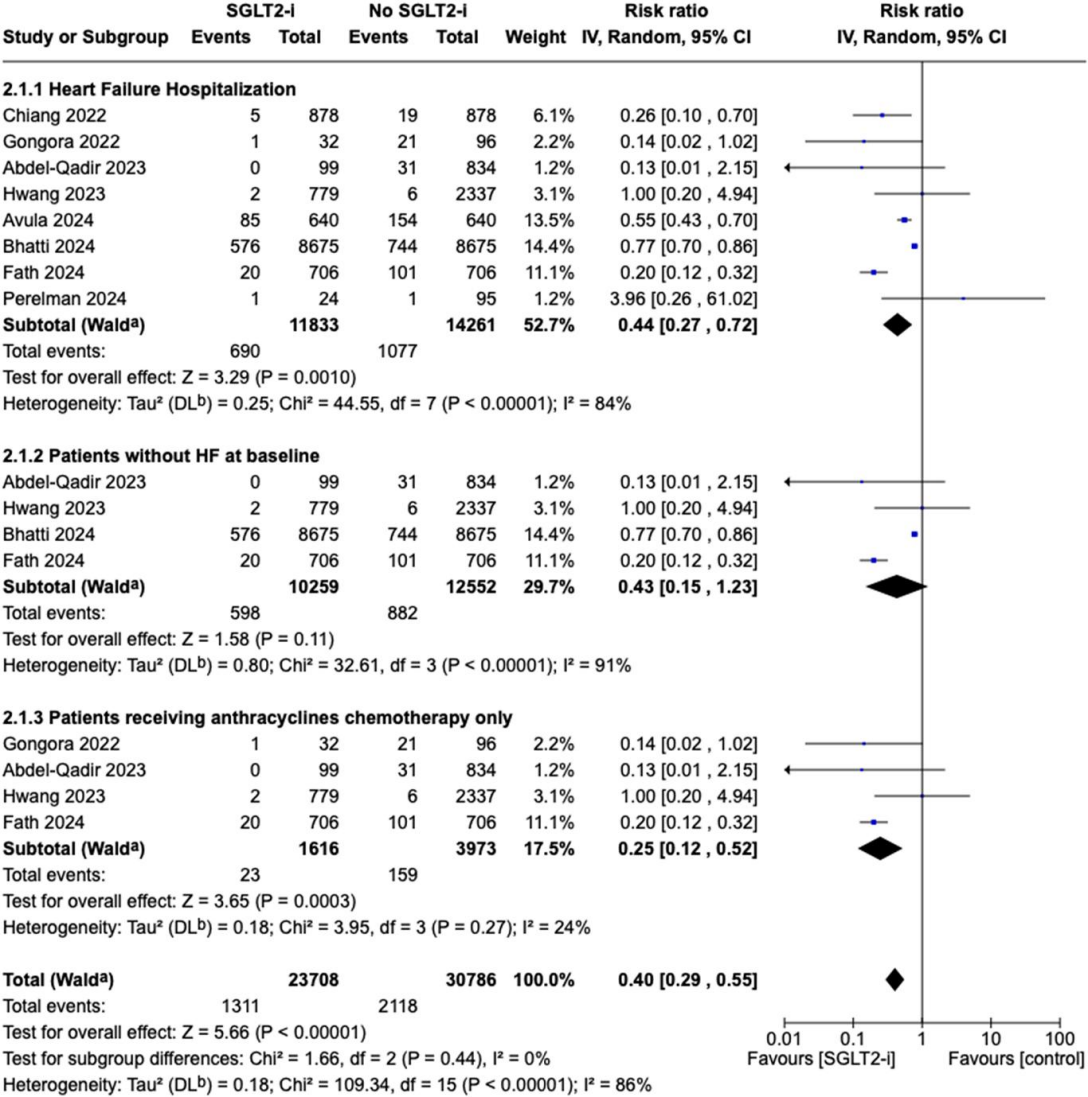
Forest plot of heart failure hospitalization. (*A*) All included studies. (*B*) Subgroup analysis of all-cause mortality in patients without heart failure at baseline. (*C*) Subgroup analysis of patients receiving anthracyclines chemotherapy only.

Eight studies reported hospitalization due to HF. Patients who used SGLT2i had a significantly lower risk of HHF than those without SGLT2i (RR 0.44, 95% CI 0.27–0.72, *P* = 0.001). The observed heterogeneity was high (*I*^2^ = 84%) (*Figure 4*). Subgroup analysis limited to patients without HF at baseline did not reach statistical significance towards reduced HHF in the SGLT2i user group (RR 0.43, 95% CI 0.15–1.23, *P* = 0.11, *I*²=91%) (*Figure 4*). Subgroup analysis restricted to studies with only anthracycline chemotherapy showed a significantly lower risk of HHF in patients who used SGLT2i with low heterogeneity (RR 0.25, 95% CI 0.12–0.52, *P* = 0.0003; *I*^2^ = 24%) (*Figure 4*). After sensitivity analysis, including five of the eight selected studies, SGLT2i use was associated with a significantly lower risk of HHF compared with non-use (RR 0.49, 95% CI 0.26–0.91, *P* = 0.03) with low heterogeneity (*I*^2^ = 38%) (see Supplementary material online, *Figure S2*).

### Publication bias

The funnel plot of primary outcomes estimates appeared asymmetric, suggesting possible publication bias (see Supplementary material online, *Figure S3*). This asymmetry may indicate that studies with negative or non-significant findings are underrepresented in the literature, potentially leading to an overestimation of the cardioprotective effects of SGLT2i. A sensitivity analysis was performed to address this concern, excluding studies with shorter follow-up durations and those with significant baseline differences in patient characteristics. However, it is important to note that asymmetry in the funnel plot could also arise from factors such as study heterogeneity or small sample sizes, rather than publication bias alone. Therefore, the results should be interpreted with caution, and future research should aim to minimize these biases.

## Discussion

This systematic review and meta-analysis offer novel insights into the intersection of cancer, cardiotoxicity, and SGLT2i in a large sample size from 11 retrospective cohort studies of diabetic patients with cancer. We have shown that SGLT2i are associated with a 53% reduction in the risk of all-cause mortality and a 56% reduction in HHF compared with patients not receiving SGLT2i in patients with cancer and Type 2 diabetes mellitus undergoing cancer therapy. The decrease in all-cause mortality and HHF remained consistent across subgroup and sensitivity analyses, reinforcing the robustness of the findings. A previous meta-analysis evaluated the CV outcomes of SGLT2i in cancer patients with diabetes; however, it included a smaller number of patients and did not assess specific subgroups. Our meta-analysis expands on this by including a larger patient population, applying stricter inclusion criteria, and performing dedicated subgroup analyses, particularly focusing on patients without baseline HF and those treated with anthracyclines.^33^

Several RCTs showed the CV and renal benefits of SGLT2i in patients with diabetes. EMPA-REG OUTCOME showed that in 7020 diabetic patients, empagliflozin use was significantly associated with lower rates of death from CV causes (HR 0.62; 95% CI 0.49–0.77; *P* < 0.001), HHF (HR 0.65; 95% CI 0.50–0.85; *P* = 0.002), and death from any cause (HR 0.68; 95% CI 0.57–0.82; *P* < 0.001).^34^ The CANVAS programme found that canagliflozin reduced the risk of CV events and HF hospitalizations in patients with Type 2 diabetes and high CV risk (HR 0.86; 95% CI 0.75–0.97; *P* < 0.001).^35^ Similarly, dapagliflozin reduced the risk of hospitalization for HF and CV death in patients with Type 2 diabetes and established CV disease (HR 0.83; 95% CI 0.73–0.95; *P* = 0.005).^36^ The use of SGLT2i has also provided encouraging results in treating patients with transthyretin cardiac amyloidosis, showing to improve prognosis.^37^

However, unlike these landmark RCTs, our meta-analysis did not show a significant reduction in the risk of HHF in patients without HF at baseline. This discrepancy may be attributed not only to the shorter follow-up duration of the included studies but also to the unique characteristics of the cancer population. In cancer patients, estimating CV risk is further complicated by the problem of competing risks. Many individuals in this cohort face high mortality rates due to cancer progression and related complications, which may mask or attenuate the detection of CV benefits.

This highlights a fundamental challenge in cardio-oncology, where clinical evidence remains limited, and uncertainties persist. Preclinical studies suggest that the potential mechanisms underlying SGLT2i cardioprotection may involve anti-inflammatory and antioxidant effects, ER stress mitigation, ketogenesis, enhanced energy metabolism, autophagy, inhibition of ferroptosis, and inhibition of ET-1.^13,18^ These have provided evidence of potential antitumour effects of SGLT2i in both experimental cell lines and animal models, and now, several clinical RCTs are investigating the antitumour effects of SGLT2i.^18,38,39^

In terms of clinical studies, the EMPACARD-PILOT trial, conducted on 76 patients, is the first and only prospective study to suggest that empagliflozin at 6 months of follow-up may reduce anthracycline-induced CTRCD.^4,16^

Other observational studies have demonstrated the cardioprotective effects of SGLT2i with a lower incidence of all-cause mortality, HF, cardiomyopathy, and arrhythmias, as well as a reduced risk of acute myocardial infarction and ischaemic stroke in cancer patients, particularly those undergoing anthracycline therapy also with extended follow-ups of 4.5–4.8 years.^23^ The use of SGLT2i did not lead to an increased risk of metastatic cancer, need for systemic antineoplastic therapy, or incidence of adverse events, reinforcing their safety profile,^24,30^ Even in the studies evaluated by our meta-analysis, the incidence of adverse events in the SGLT2i group was similar to that of the control group. According to the literature, genitourinary infection is the most common adverse event followed by hypoglycaemia, especially if in combination with metformin and much more rarely acute renal failure, ketoacidosis, hyperosmolar hyperglycaemic state or hyperglycaemia, and Fournier’s gangrene.

At present, it remains unclear whether SGLT2i directly decrease the risk of mortality and HHF in patients with cancer, or whether SGLT2i use is a marker of better performance status or better overall conditions, which makes more likely the prescription of non-oncological drugs.

In the studies included in our analysis, all-cause mortality is often used as the primary outcome; however, its relevance in cancer patients is questionable. In cancer populations, distinguishing whether death results from CV problems or cancer progression is complex and often unclear, as underlying disease and treatment side effects can confound the characterization of CV causes. Only one study explicitly separates cancer-related mortality from all-cause mortality, highlighting the need for more accurate outcome reporting to interpret mortality factors in these patients.^23^ The continued exclusion of active cancer patients from major CV RCTs does not help to clarify the role of these drugs in primary prevention, underscoring an urgent need for studies that specifically address this vulnerable population. Some RCTs are currently in the recruitment phase to evaluate the role of these drugs in CTRCD (NCT06341842, NCT06427226) undergoing anthracycline treatment (NCT06304857, NCT05271162, NCT06103279).

Such studies will be crucial to uncover the underlying mechanisms of SGLT2i in the context of cancer and establish clearer evidence of their safety and efficacy. Understanding the interaction of SGLT2i with CVD and cancer treatments will enable more effective and personalized CV care in this high-risk group.

### Limitations

All included studies were observational, making it impossible to exclude potential confounders and unrecognized factors, such as better overall health status or socioeconomic conditions, which could influence outcomes. There was variability in SGLT2i type, dosage, and treatment duration, and follow-up periods ranged from 1 to 4.8 years. This may be insufficient to assess long-term safety and efficacy, especially regarding the late cardiotoxic effects of anthracyclines. The primary endpoints varied across studies, and high heterogeneity in all-cause mortality analysis may reflect differences in patient populations, cancer types, and treatment regimens. Furthermore, the included studies did not distinguish between different causes of mortality, making it unclear whether the observed reduction in all-cause mortality is primarily driven by the metabolic effects of SGLT2i in diabetes management or by their broader cardioprotective benefits in cancer patients.

Despite subgroup and sensitivity analyses, these factors remain relevant. Uric acid (UA) is often elevated in cancer patients, likely due to cancer-related cell turnover and the effects of antitumour therapies.^40^ Although SGLT2i have been shown to reduce UA levels in other populations, their specific role and CV effects in cancer patients remains unexplored in the included studies. Additionally, five studies reported event numbers only after propensity score matching, potentially impacting the overall analysis.

## Conclusions

This meta-analysis shows that SGLT2i use may be associated with a significant reduction in all-cause mortality and risk of HF hospitalization in cancer patients with Type 2 diabetes. Randomized controlled trials are needed to confirm the cardioprotective effects of SGLT2i in cancer patients, both with and without diabetes, and to assess their safety and efficacy across different cancer types and treatment protocols. Sodium–glucose cotransporter 2 inhibitors may offer a promising strategy to improve CV health and survival in this high-risk population.

### Perspectives

This meta-analysis highlights the potential cardioprotective effects of SGLT2i in cancer patients with Type 2 diabetes mellitus undergoing chemotherapy, particularly in reducing all-cause mortality and HF hospitalizations. However, the lack of RCTs in this population necessitates further investigation. Future studies should clarify the direct cardioprotective mechanisms of SGLT2i, beyond their metabolic effects, and identify the patient subgroups that benefit most.^41^ Specifically, prospective RCTs are needed in cancer patients both with and without diabetes, particularly those at high risk of cardiotoxicity, to fully assess their potential protective role against anthracycline-induced cardiac dysfunction. Research should also focus on the molecular pathways involved, such as anti-inflammatory and antifibrotic effects, and assess their role in preventing late-onset cardiotoxicity in cancer survivors. Integrating SGLT2i into cardio-oncology care could transform CV risk management in this population, particularly during cardiotoxic treatments. Multidisciplinary efforts will be key to translating these findings into practice.

## Supplementary Material

pvaf028_Supplementary_Data

## Data Availability

The data underlying this article will be shared on reasonable request to the corresponding author.

## References

[1] Murphy CC, Gerber DE, Pruitt SL. Prevalence of prior cancer among persons newly diagnosed with cancer: an initial report from the surveillance, epidemiology, and End results program. JAMA Oncol 2018;4:832–836. 10.1001/jamaoncol.2017.3605 29167866 PMC6370034

[2] Yeh ETH, Bickford CL. Cardiovascular complications of cancer therapy: incidence, pathogenesis, diagnosis, and management. J Am Coll Cardiol 2009;53:2231–2247. 10.1016/j.jacc.2009.02.050 19520246

[3] Cardinale D, Iacopo F, Cipolla CM. Cardiotoxicity of anthracyclines. Front Cardiovasc Med 2020;7:26. 10.3389/fcvm.2020.00026 32258060 PMC7093379

[4] Lyon AR, López-Fernández T, Couch LS, Asteggiano R, Aznar MC, Bergler-Klein J, Boriani G, Cardinale D, Cordoba R, Cosyns B, Cutter DJ, de Azambuja E, de Boer RA, Dent SF, Farmakis D, Gevaert SA, Gorog DA, Herrmann J, Lenihan D, Moslehi J, Moura B, Salinger SS, Stephens R, Suter TM, Szmit S, Tamargo J, Thavendiranathan P, Tocchetti CG, van der Meer P, van der Pal HJH; ESC Scientific Document Group. 2022 ESC guidelines on cardio-oncology developed in collaboration with the European Hematology Association (EHA), the European Society for Therapeutic Radiology and Oncology (ESTRO) and the International Cardio-Oncology Society (IC-OS). Eur Heart J 2022;43:4229–4361. 10.1093/eurheartj/ehac244 36017568

[5] Cardinale D, Colombo A, Lamantia G, Colombo N, Civelli M, De Giacomi G, Rubino M, Veglia F, Fiorentini C, Cipolla CM. Anthracycline-induced cardiomyopathy: clinical relevance and response to pharmacologic therapy. J Am Coll Cardiol 2010;55:213–220. 10.1016/j.jacc.2009.03.095 20117401

[6] Cardinale D, Colombo A, Bacchiani G, Tedeschi I, Meroni CA, Veglia F, Civelli M, Lamantia G, Colombo N, Curigliano G, Fiorentini C, Cipolla CM. Early detection of anthracycline cardiotoxicity and improvement with heart failure therapy. Circulation 2015;131:1981–1988. 10.1161/CIRCULATIONAHA.114.013777 25948538

[7] Jannazzo A, Hoffman J, Lutz M. Monitoring of anthracycline-induced cardiotoxicity. Ann Pharmacother 2008;42:99–104. 10.1345/aph.1K359 18094345

[8] Sławiński G, Hawryszko M, Liżewska-Springer A, Nabiałek-Trojanowska I, Lewicka E. Global longitudinal strain in cardio-oncology: a review. Cancers (Basel) 2023;15:986. 10.3390/cancers15030986 36765941 PMC9913863

[9] Di Lisi D, Madaudo C, Ortello A, Rubino L, Scelfo D, Sinagra FP, Comparato F, Triolo OF, Rossetto L, Galassi AR, Novo G. Assessment of cancer therapy-related cardiac dysfunction in breast cancer women using a new speckle tracking echocardiography Index: the GAVS. Echocardiography 2024;41:e15881. 10.1111/echo.15881 39007868

[10] Di Lisi D, Manno G, Madaudo C, Filorizzo C, Intravaia RCM, Galassi AR, Incorvaia L, Russo A, Novo G. Chemotherapy-related cardiac dysfunction: the usefulness of myocardial work indices. Int J Cardiovasc Imaging 2023;39:1845–1853. 10.1007/s10554-023-02897-9 37548845

[11] Di Lisi D, Moreo A, Casavecchia G, Cadeddu Dessalvi C, Bergamini C, Zito C, Madaudo C, Madonna R, Cameli M, Novo G. Atrial strain assessment for the early detection of cancer therapy-related cardiac dysfunction in breast cancer women (the STRANO STUDY: atrial strain in cardio-oncology). J Clin Med 2023;12:7127. 10.3390/jcm12227127 38002739 PMC10672006

[12] Wright EM . SGLT2 inhibitors: physiology and pharmacology. Kidney360 2021;2:2027–2037. 10.34067/KID.0002772021 35419546 PMC8986039

[13] Lopaschuk GD, Verma S. Mechanisms of cardiovascular benefits of sodium glucose co-transporter 2 (SGLT2) inhibitors: a state-of-the-art review. JACC Basic Transl Sci 2020;5:632–644. 10.1016/j.jacbts.2020.02.004 32613148 PMC7315190

[14] Vaduganathan M, Docherty KF, Claggett BL, Jhund PS, de Boer RA, Hernandez AF, Inzucchi SE, Kosiborod MN, Lam CSP, Martinez F, Shah SJ, Desai AS, McMurray JJV, Solomon SD. SGLT2 inhibitors in patients with heart failure: a comprehensive meta-analysis of five randomised controlled trials. Lancet 2022;400:757–767. 10.1016/S0140-6736(22)01429-5 36041474

[15] Packer M, Butler J, Zannad F, Filippatos G, Ferreira JP, Pocock SJ, Carson P, Anand I, Doehner W, Haass M, Komajda M, Miller A, Pehrson S, Teerlink JR, Schnaidt S, Zeller C, Schnee JM, Anker SD. Effect of empagliflozin on worsening heart failure events in patients with heart failure and preserved ejection fraction: EMPEROR-preserved trial. Circulation 2021;144:1284–1294. 10.1161/CIRCULATIONAHA.121.056824 34459213 PMC8522627

[16] Daniele AJ, Gregorietti V, Costa D, López-Fernández T. Use of EMPAgliflozin in the prevention of CARDiotoxicity: the EMPACARD—PILOT trial. Cardio-Oncol. Lond. Engl 2024;10:58. 10.1186/s40959-024-00260-y PMC11375926 39237985

[17] Mohite P, Lokwani DK, Sakle NS. Exploring the therapeutic potential of SGLT2 inhibitors in cancer treatment: integrating in silico and in vitro investigations. Naunyn. Schmiedebergs Arch. Pharmacol 2024;397:6107–6119. 10.1007/s00210-024-03021-x 38416196

[18] Dabour MS, George MY, Daniel MR, Blaes AH, Zordoky BN. The cardioprotective and anticancer effects of SGLT2 inhibitors. JACC CardioOncol 2024;6:159–182. 10.1016/j.jaccao.2024.01.007 38774006 PMC11103046

[19] Liu L, Chen HH, Kuo HH, Lin PL. Effects of SGLT2 inhibitors on the cardiovascular outcomes in patients with cancer: a systematic review and meta-analysis. Eur Heart J 2024;45:ehae666.3164. 10.1093/eurheartj/ehae666.3164

[20] Page MJ, McKenzie JE, Bossuyt PM, Boutron I, Hoffmann TC, Mulrow CD, Shamseer L, Tetzlaff JM, Akl EA, Brennan SE, Chou R, Glanville J, Grimshaw JM, Hróbjartsson A, Lalu MM, Li T, Loder EW, Mayo-Wilson E, McDonald S, McGuinness LA, Stewart LA, Thomas J, Tricco AC, Welch VA, Whiting P, Moher D. The PRISMA 2020 statement: an updated guideline for reporting systematic reviews. PLoS Med 2021;18:e1003583. 10.1371/journal.pmed.1003583 33780438 PMC8007028

[21] Hendryx M, Dong Y, Ndeke JM, Luo J. Sodium-glucose cotransporter 2 (SGLT2) inhibitor initiation and hepatocellular carcinoma prognosis. PLoS One 2022;17:e0274519. 10.1371/journal.pone.0274519 36094949 PMC9467321

[22] Luo J, Hendryx M, Dong Y. Sodium-glucose cotransporter 2 (SGLT2) inhibitors and non-small cell lung cancer survival. Br J Cancer 2023;128:1541–1547. 10.1038/s41416-023-02177-2 36765176 PMC10070339

[23] Huang Y-M, Chen W-M, Jao A-T, Chen M, Shia B-C, Wu S-Y. Effects of SGLT2 inhibitors on clinical cancer survival in patients with type 2 diabetes. Diabetes Metab 2024;50:101500. 10.1016/j.diabet.2023.101500 38036054

[24] Bhatti AW, Patel R, Dani SS, Khadke S, Makwana B, Lessey C, Shah J, Al-Husami Z, Yang EH, Thavendiranathan P, Neilan TG, Sadler D, Cheng RK, Dent SF, Liu J, Lopez-Fernandez T, Herrmann J, Scherrer-Crosbie M, Lenihan DJ, Hayek SS, Ky B, Deswal A, Barac A, Nohria A, Ganatra S. SGLT2i and primary prevention of cancer therapy-related cardiac dysfunction in patients with diabetes. JACC CardioOncol 2024;6:863–875. 10.1016/j.jaccao.2024.08.001 39801650 PMC11711834

[25] Fath AR, Aglan M, Aglan A, Chilton RJ, Trakhtenbroit A, Al-Shammary OA, Oppong-Nkrumah O, Lenihan DJ, Dent SF, Otchere P. Cardioprotective potential of sodium-glucose cotransporter-2 inhibitors in patients with cancer treated with anthracyclines: an observational study. Am J Cardiol 2024;222:175–182. 10.1016/j.amjcard.2024.04.032 38692401

[26] Abdel-Qadir H, Carrasco R, Austin PC, Chen Y, Zhou L, Fang J, Su HMH, Lega IC, Kaul P, Neilan TG, Thavendiranathan P. The association of sodium-glucose cotransporter 2 inhibitors with cardiovascular outcomes in anthracycline-treated patients with cancer. JACC CardioOncol 2023;5:318–328. 10.1016/j.jaccao.2023.03.011 37397088 PMC10308059

[27] Higgins JPT, Thompson SG, Deeks JJ, Altman DG. Measuring inconsistency in meta-analyses. BMJ 2003;327:557–560. 12958120 10.1136/bmj.327.7414.557 PMC192859

[28] Gongora CA, Drobni ZD, Quinaglia Araujo Costa Silva T, Zafar A, Gong J, Zlotoff DA, Gilman HK, Hartmann SE, Sama S, Nikolaidou S, Suero-Abreu GA, Jacobsen E, Abramson JS, Hochberg E, Barnes J, Armand P, Thavendiranathan P, Nohria A, Neilan TG. Sodium-glucose co-transporter-2 inhibitors and cardiac outcomes among patients treated with anthracyclines. JACC Heart Fail 2022;10:559–567. 10.1016/j.jchf.2022.03.006 35902159 PMC9638993

[29] Chiang CH, Chiang CH, Chiang CH, Ma KS, Peng CY, Hsia YP, Horng CS, Chen CY, Chang YC, See XY, Chen YJ, Wang SS, Suero-Abreu GA, Peterson LR, Thavendiranathan P, Armand P, Peng CM, Shiah HS, Neilan TG. Impact of sodium-glucose cotransporter-2 inhibitors on heart failure and mortality in patients with cancer. Heart 2023;109:470–477. 10.1136/heartjnl-2022-321545 36351793 PMC10037540

[30] Hwang H-J, Kim M, Jun JE, Yon DK. Sodium-glucose cotransporter-2 inhibitors improve clinical outcomes in patients with type 2 diabetes Mellitus undergoing anthracycline-containing chemotherapy: an emulated target trial using nationwide cohort data in South Korea. Sci Rep 2023;13:21756. 10.1038/s41598-023-48678-1 38066029 PMC10709414

[31] Avula V, Sharma G, Kosiborod MN, Vaduganathan M, Neilan TG, Lopez T, Dent S, Baldassarre L, Scherrer-Crosbie M, Barac A, Liu J, Deswal A, Khadke S, Yang EH, Ky B, Lenihan D, Nohria A, Dani SS, Ganatra S. SGLT2 inhibitor use and risk of clinical events in patients with cancer therapy–related cardiac dysfunction. JACC Heart Fail 2024;12:67–78. 10.1016/j.jchf.2023.08.026 37897456

[32] Perelman MG, Brzezinski RY, Waissengrin B, Leshem Y, Bainhoren O, Rubinstein TA, Perelman M, Rozenbaum Z, Havakuk O, Topilsky Y, Banai S, Wolf I, Laufer-Perl M. Sodium-glucose co-transporter-2 inhibitors in patients treated with immune checkpoint inhibitors. Cardio-Oncol 2024;10:2. 10.1186/s40959-023-00199-6 PMC10782769 38212825

[33] Kuo HH, Wang KT, Chen HH, Lai ZY, Lin PL, Chuang YJ, Liu LY. Cardiovascular outcomes associated with SGLT2 inhibitor therapy in patients with type 2 diabetes Mellitus and cancer: a systematic review and meta-analysis. Diabetol Metab Syndr 2024;16:108. 10.1186/s13098-024-01354-4 38773486 PMC11110336

[34] Zinman B, Wanner C, Lachin JM, Fitchett D, Bluhmki E, Hantel S, Mattheus M, Devins T, Johansen OE, Woerle HJ, Broedl UC, Inzucchi SE; EMPA-REG OUTCOME Investigators. Empagliflozin, cardiovascular outcomes, and mortality in type 2 diabetes. N Engl J Med 2015;373:2117–2128. 10.1056/NEJMoa1504720 26378978

[35] Neal B, Perkovic V, Mahaffey KW, De Zeeuw D, Fulcher G, Erondu N, Shaw W, Law G, Desai M, Matthews DR. Canagliflozin and cardiovascular and renal events in type 2 diabetes. N Engl J Med 2017;377:644–657. 10.1056/NEJMoa1611925 28605608

[36] Wiviott SD, Raz I, Bonaca MP, Mosenzon O, Kato ET, Cahn A, Silverman MG, Zelniker TA, Kuder JF, Murphy SA, Bhatt DL, Leiter LA, McGuire DK, Wilding JPH, Ruff CT, Gause-Nilsson IAM, Fredriksson M, Johansson PA, Langkilde AM, Sabatine MS; DECLARE–TIMI 58 Investigators. Dapagliflozin and cardiovascular outcomes in type 2 diabetes. N Engl J Med 2019;380:347–357. 10.1056/NEJMoa1812389 30415602

[37] Karakasis P, Theofilis P, Patoulias D, Schuermans A, Vlachakis PK, Klisic A, Rizzo M, Fragakis N. Sodium-glucose cotransporter 2 inhibitors and outcomes in transthyretin amyloid cardiomyopathy: systematic review and meta-analysis. Eur J Clin Invest 2025; 10.1111/eci.14392 PMC12066899 39868862

[38] Papadopoli D, Uchenunu O, Palia R, Chekkal N, Hulea L, Topisirovic I, Pollak M, St-Pierre J. Perturbations of cancer cell metabolism by the antidiabetic drug canagliflozin. Neoplasia 2021;23:391–399. 10.1016/j.neo.2021.02.003 33784591 PMC8027095

[39] Zhou J, Zhu J, Yu SJ, Ma HL, Chen J, Ding XF, Chen G, Liang Y, Zhang Q. Sodium-glucose co-transporter-2 (SGLT-2) inhibition reduces glucose uptake to induce breast cancer cell growth arrest through AMPK/mTOR pathway. Biomed Pharmacother 2020;132:110821. 10.1016/j.biopha.2020.110821 33068934

[40] Packer M . Hyperuricemia and gout reduction by SGLT2 inhibitors in diabetes and heart failure. J Am Coll Cardiol 2024;83:371–381. 10.1016/j.jacc.2023.10.030 38199714

[41] Camilli M, Viscovo M, Maggio L, Bonanni A, Torre I, Pellegrino C, Lamendola P, Tinti L, Teofili L, Hohaus S, Lanza GA, Ferdinandy P, Varga Z, Crea F, Lombardo A, Minotti G. Sodium–glucose cotransporter 2 inhibitors and the cancer patient: from diabetes to cardioprotection and beyond. Basic Res Cardiol 2024;120:241–262. 10.1007/s00395-024-01059-9 38935171 PMC11790819

