# Supplementary material for: Effects of sodium–glucose cotransporter 2 inhibitors in patients with cancer and diabetes mellitus: a systematic review and meta-analysis
Source: Eur Heart J Cardiovasc Pharmacother. 2025 Apr 25;11(4):343–52. doi: 10.1093/ehjcvp/pvaf028 (PMC12231130; doi:10.1093/ehjcvp/pvaf028)
Supplement: pvaf028_Supplementary_Data [file pvaf028_supplementary_data.zip › Supplementary Files.docx]

**Supplementary Tables**

|  | 1 | Diabetes Mellitus/ or diabet*.mp. |
| --- | --- | --- |
|  | 2 | Neoplasms/ or cancer*.mp. or neoplasm*.mp. |
|  | 3 | Cardiotoxicity/ or cardiotoxic*.mp. |
|  | 4 | SGLT2 Inhibitors/ or SGLT2.mp. or Sodium-Glucose Transporter 2 Inhibitors.mp |
|  | 5 | 1 and 2 and 3 and 4 |
|  | 6 | limit 5 to english language |
|  | 7 | limit 6 to humans |
|  |  | (("Diabetes Mellitus"[MeSH] OR diabet*.mp.) AND ("Neoplasms"[MeSH] OR cancer*.mp. OR neoplasm*.mp.) AND ("Cardiotoxicity"[MeSH] OR cardiotoxic*.mp.) AND ("Sodium-Glucose Transporter 2 Inhibitors"[MeSH] OR SGLT2.mp.)). |
|  | | |

**Supplementary Table 1.** Research strategy in PubMed, OVID, Medline, Embase, Web of Science, and the Cochrane Library and MeSH Strategy on PubMed.

**Supplementary Table 2:** Full details of baseline characteristics of the selected studies.

**Supplementary Figures**

**
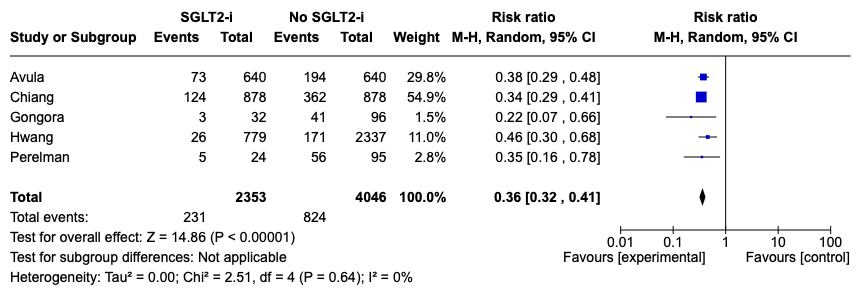
 Supplementary Figure 1.** Forest plot of all-cause mortality after sensitivity analysis, excluding studies with unspecified chemotherapy regimens, the study with the shortest follow-up (1 year), and studies with significant baseline population differences or conflicting definitions of outcomes.

**
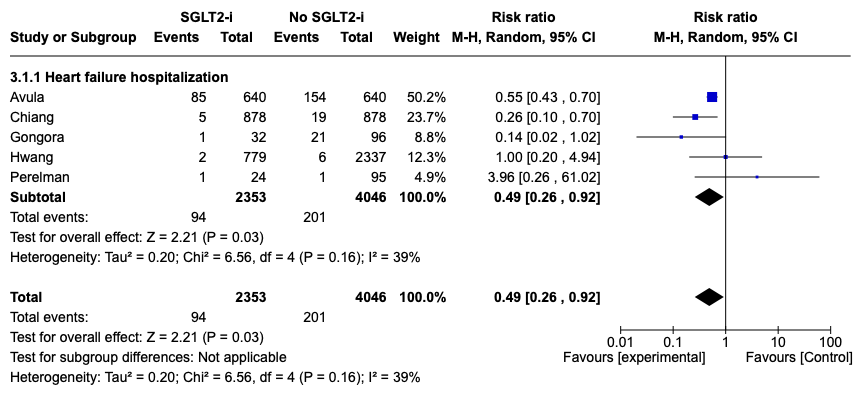
**

**Supplementary Figure 2.** Forest plot of heart failure hospitalization after sensitivity analysis, excluding studies with unspecified chemotherapy regimens, the study with the shortest follow-up (1 year), and studies with significant baseline population differences or conflicting definitions of outcomes.

**Supplementary Figure 3:** Funnel Plot of primary outcomes.
