# Supplementary material for: Effects of sodium–glucose cotransporter 2 inhibitors in patients with cancer and diabetes mellitus: a systematic review and meta-analysis
Source: Eur Heart J Cardiovasc Pharmacother. 2025 Apr 25;11(4):343–52. doi: 10.1093/ehjcvp/pvaf028 (PMC12231130; doi:10.1093/ehjcvp/pvaf028)
Supplement: pvaf028_Supplementary_Data [file pvaf028_supplementary_data.zip › Supplementary Table 2.docx]

| First Author | Year of publication | N. Of Population | | Population based | Inclusion Criteria | Esclusion Criteria | HF Patients | LVEF% | Cancers | Chemotherapy | Outcomes | Follow-up | Adverse Events | Interpretation |
| --- | --- | --- | --- | --- | --- | --- | --- | --- | --- | --- | --- | --- | --- | --- |
| Gongora | 2022 | 32 patients treated with SGLT2i  **empagliflozin** (50%),  **canagliflozin** (34%),  **dapagliflozin** (16%) | 96 patients treated without SGLT2i | U.S.A | Diabetes  History of cancer  Prior diagnoses of HF  Treated before Sept 2020 | Diabetes coded after antracycline initiation  < 18 years | 9 (13%) | SGLT2i  Group: 62 ± 5  No SGLT2i  Group: 64 ± 7 | Lymphoma 44 (34%)  Breast 31 (24%)  Genitourinary 21 (16%)  Gastrointestinal 12 (9%)  Sarcoma 9 (7%)  Leukemia 4 (3%)  Other cancers 7 (5%) | Antracyclines - Doxorubicin | **Primary Outcome:** composite consisting of HF incidence, HF ad-  missions, the development of cardiomyopathy  (defined as a >10% decline in left ventricular ejection  fraction to <53%) or clinically significant arrhythmias  **Primary safety outcome**  all-cause  mortality  **Secondary Outcome:**  composite of sepsis and neutropenic fever. | 1.5 years | Genital yeast infections (Minor increase) | SGLT2 inhibitors was associated with a  lower rate of cardiac events after anthracycline ther-  apy in patients with cancer and DM |
| Chiang | 2022 | 878 patients treated with SGLT2i  **empagliflozin (49%),**  **dapagliflozin (38%)** | 878 patients treated without SGLT2i | Taiwan | Diabetes  History of cancer  Treated between 2010-2021 | Patients with only one hospital visit and missing data  < 18 years | 84 (5%) | Not specified | Gastrointestinal 623 (35%)  Genitourinary 311 (18%)  Thoracic 222 (13%)  Head and neck 180 (10%)  Breast 199 (11%)  Haematological 83 (5%)  Skin 27 (2%)  Others 111 (6%) | Anthracyclines 136 (8%)  Tyrosine kinase inhibitors 71 (4%)  Radiotherapy 47 (3%)  Alkylating agents 82 (5%)  Antimetabolites 310 (18%)  Platinum 216 (12%)  Plant alkaloids 190 (11%)  HER2 inhibitors 32 (2%)  VEGF inhibitors 29 (2%)  Immune checkpoint inhibitors 22 (1%) | **Primary Outcome:** hospitalisation for incident HF  **Primary safety outcome**  all-cause  Mortality  **Secondary Outcome:**  serious  adverse events reported as associated with the use of SGLT2i,  including diabetic ketoacidosis, urosepsis, sepsis, hypogly-  caemia, acute kidney injury and Fournier’s gangrene | 1.6 years | Fournier’s gangrene (One patient) | SGLT2i  was associated with a reduction in hospitalisation for HF and  improved overall survival among patients with cancer with type  2 diabetes mellitus. There were no increased rates of serious  adverse events associated with SGLT2i. |
| Hendrix | 2022 | 137 patients treated with SGLT2i  Types of SGLT2-i not specified | 3048  patients treated without SGLT2i | U.S.A | Diabetes  Diagnosis of hepatocellular carcinoma between 2014 and 2017 | Patients with no HMO coverage for at least 12 months prior to  cancer diagnosis and 3 months after cancer diagnosis | Not specified | Not specified | Hepatocellular carcinoma (HCC) 3185 100% | Chemotherapy 1274 (40%) – Not Specified  Radiotherapy 807 (25.34%) | **Primary Outcome:** All-cause mortality | 1.8 years | Not specified | HCC  patients with pre-existing T2DM treated with SGLT2 inhibitors had significantly lower risk of mortality, especially among those treated more than 12 months. |
| Abdel-Qadir | 2023 | 99 patients treated with SGLT2i  Types of SGLT2-i not specified | 834 patients treated without SGLT2i | Canada | Diabetes  History of cancer who received anthracycline-based  ≥65 years  Treated Jan 2016- Dec 2019 | Prior diagnoses of HF | 0 (0%) | Not specified | Breast 322 (34.5%)  Lymphnode 263 (28.2%)  Stomach 59 (6.3%)  Bone marrow 56 (6.0%)  Ovary 43 (4.6%)  Lip, oral cavity, pharynx, nasal cavity, accessory sinuses 28 (3.0%)  Musculoskeletal or soft tissue 27 (2.9%)  Urinary tract 19 (2.0%)  Skin 16 (1.7%)  Unknown primary site 13 (1.4%)  Bronchus and lung 12 (1.3%)  Gastrointestinal site 12 (1.3%)  Liver and intrahepatic bile ducts 7 (0.8%)  Uterus 7 (0.8%)  Colorectal 7 (0.8%)  Thyroid gland 6 (0.6%) | Antracyclines | **Primary Outcome:**  composite of newly incident HF, HF admissions, clinically  significant arrhythmias, or a >10% absolute decline in  left ventricular ejection fraction to a final value <53%.  **Secondary Outcome:**  hospitalizations that included  CVD within any of the discharge diagnostic fields | 1.6 years | <6 documented  hypoglycemia events  <6 hospitalizations with a most  responsible diagnosis related to diabetes in SGLT2i-treated  No documented episodes of diabetic  ketoacidosis, hyperosmolar hyperglycemic state, or  hyperglycemia | SGLT2is may reduce the risk of HF in patients receiving anthracycline-containing chemotherapy. We did not observe important safety concerns with concurrent exposure to SGLT2is and chemotherapy. Rather, we observed a lower rate of death associated with SGLT2i use that was not statistically significant. |
| Huang | 2023 | 16711 patients treated with SGLT2i  Types of SGLT2-i not specified | 33422 patients treated without SGLT2i | Taiwan | Diabetes  History of cancer without metastasis between between January 1, 2016 and December 31, 2020  > 20 years  receipt of at least one type of antidiabetic medication  prescribed SGLT2i ≥28 or 0 cumulative defined daily doses of SGLT2is after  cancer diagnosis | History of cancer before diagnosis  of T2DM and that of synchronous or metachronous double cancers | 2533 (5%) | Not specified | Pancreatic cancer 433 (0.8%)  Hepatocellular carcinoma 4441 (8.8%)  Esophageal cancer 309 (0.6%)  Head and neck cancer 5931 (11.8%)  Gastric cancer 957 (1.90%)  Lung cancer 3684 (7.3%)  Colorectal cancer 11366(22.7%)  Gynecologic cancer 583 (11.6%)  Breast cancer 10224 (20.4%)  Prostate cancer 4510 (8.9%)  Other cancers 7695 (15.3%) | Chemotherapy – Not specified | **Primary Outcome:**  all-cause mortality  **Secondary Outcome:**  cancer-specific  mortality | 4.5/ 4.8 years | Not specified | SGLT2is may increase Overall Survival and Cancer-Specific Survival  in patients with cancer in a dose-dependent manner, regardless of patient  sex, age, cancer type, American Joint Committee on Cancer clinical  stage, adapted Diabetic Complication Severity Index score, BMI, and  cigarette smoking status. |
| Luo | 2023 | 531 patients treated with SGLT2i  Types of SGLT2-i not specified | 24384 patients treated without SGLT2i | U.S.A | Diabetes  Non-small cell lung cancer cancer as a  primary cancer between 2014 and 2017 | Patients with no HMO coverage for at least 12 months prior to  cancer diagnosis and 3 months after cancer diagnosis diagnosis or until death | Not specified | Not specified | Non-small cell lung cancer 24915 (100%) | Chemotherapy – Not specified 9392 (37.70%)  Radiation 11221 (45.04%)  Immunotherapy 1071 (4.3%)  EGFR Antagonist 971 (3.9%) | **Primary Outcome:**  All-cause mortality | 1.3 years | Not specified | SGLT2 inhibitors use  appeared to be bene cial for prolonged NSCLC survivorship  among people with type 2 diabetes. |
| Hwang | 2023 | 779 patients treated with SGLT2i  Types of SGLT2-i not specified | 2337 patients treated without SGLT2i | South Korea | Diabetes  ≥ 18 years  New diagnosis of  cancer and underwent AC-containing chemotherapy between January 2014 and December 2021 | Patients diagnosed with cancer in 2013 or earlier;  Metastasis  or malignancy on other sites on the index date;  Patients with preexisting significant cardiac diseases, including  cardiac arrest, myocardial infarction or ischemic heart disease, **HF** or cardiomyopathy, cardiac valve diseases,  and atrial and ventricular arrhythmias;  Patients with a history of severe or decompensated systemic diseases,  including severe lung diseases, end-stage renal diseases or dialysis, and liver cirrhosis or hepatic failure; patients  with preexisting stroke;  Patients with type 1 DM; users of insulin or glucagon-like peptide-1 receptor agonists;  and patients with change of SGLT2i within four months from the index date. | 0 (0%) | Not specified | Lymphoma 614 (15%)  Breast 2,096 (49%)  Genitourinary 421 (10%)  Other cancers 1126 (27%) | Doxorubicin 3981 (94%)  Epirubicin 248 (5%)  Doxorubicin + Epirubicin 6 (1%)  High-dose ACs 1346 (32%)  Alkylating agents 140 (3%)  Antimicrotubule agents 1383 (33%)  HER2 inhibitors 474 (11%)  VEGF-targeting agents 224 (5%) | **Primary Outcome:** HF hospitalization, AMI, ischemic stroke, death,  **Secondary Outcome:**  the composite outcome of HF hospitalization, AMI, ischemic stroke, and death | 3.4 years | Not specified | GLT2i may contribute to decreasing  mortality and improving clinical outcomes in patients with T2DM undergoing AC-containing chemotherapy. |
| Avula | 2024 | 640 patients treated with SGLT2i  Types of SGLT2-i not specified | 640 patients treated with contemporary HF Medications (ACEi/ARB/ARNI+Beta blockers+ MRA) without SGLT2i | U.S.A | Diabetes  History of cancer  ≥18 years  Exposure to potentially cardiotoxic antineoplastic therapies  With a subsequent diagnosis of cardiomiopathy or HF (**CTRCD**) between Jan 2013- April 2020 | Patients with diagnosis of acute coronary syndrome (AMI, STEMI, NSTEMI) or who underwent CABG or PCI after starting antineoplastic therapy  Patients without optimal GDMT (ACEi/ARB/ARNI+Beta blockers+ MRA) | 1280 (100%) | **LVEF < 40%**  SGLT2i  Group: 138 (21.6%)  No SGLT2i  Group: 127 (19.8%) | Breast 199 (15.5%)  Lymphomas 314 (24.5%)  Myelodysplastic syndromes 474 (37%)  Genitourinary 75 (6%)  Gastrointestinal 253 (20%)  Gynecologic 41 (3.2%)  Respiratory and intrathoracic organs 66 (5%)  Mesothelial and soft tissue 28 (2.2%)  Neoplasms of unspecied behavior 279 (22%)  Metastatic malignancy 382 (30%) | Alkylating agents 404 (31.5%)  Anthracenediones (Mitoxantrone) 20 (1.5%)  Anthracyclines 255 (20%)  Antimetabolites 516 (40%)  Aromatase inhibitor 92 (7%)  Monoclonal antibodies 449 (35%)  Proteosome inhibitors 85 (6.6%)  Small-molecule TKIs 235 (18.3%)  Radiation therapy 146 (11.4%) | **Primary Outcome:** HF exacerbations  And all-cause mortality  **Secondary Outcome:**  all-cause hospi-talizations or emergency department (ED) visits,  atrial fibrillation and utter, acute kidney injury, and  the need for renal replacement therapy | 2 years | Urinary tract infections  (9.2%)  Lower extremity amputations (1.6%) | In patients with  CTRCD and HF treated with SGLT2 inhibitors in  addition to other GDMTs, are associated with a lower  rate of acute HF exacerbation, all-cause mortality,  hospitalizations or ED visits, atrial fibrillation or  utter, acute kidney injury, and renal replacement  therapy when compared with patients on contemporary GDMT except for SGLT2 inhibitors. |
| Fath | 2024 | 706 patients treated with SGLT2i  **empagliflozin** (57%),  **canagliflozin** (31%),  **dapagliflozin** (26%),  ertugliflozin (1.4%. | 706 patients treated without SGLT2i | U.S.A | ≥18 years  Diagnosis of cancer who received  anthracycline-based chemotherapy  between January 1, 2014, and December 31, 2021. | Current or previous diagnosis of HF | 0 (0%) | SGLT2i  Group: 62.8 ± 8.2  No SGLT2i  Group: 63.6 ± 9 | Hematological and lymphatic 542 (38.3%)  Breast 363 (25.7%)  Gastrointestinal and digestive organs 337 (23.8%)  Female genital organs 128 (9%)  Mesothelial and soft tissue 89 (6.3%)  Urinary tract 67 (4.7%)  Skin 65 (4.6%)  Male genital organs 51 (3.6%)  Respiratory and intrathoracic organs 38 (2.7%)  Endocrine glands 32 (2.2%)  Oropharynx 22 (1.5%)  Neuroendocrine 21 (1.5%)  Bone and cartilage 21 (1.5%)  Central nervous system 20 (1.4%)  Other secondary and unspecified sites 446 (31.6%) | Anthracyclines 1412 (100%)  Specifically:  Doxorubicin 1221 (96%)  Daunorubicin 72 (5.15)  Epirubicin 63 (4.45%)  Idarubicin 51 (3.6%)  Valrubicin 20 (1.4%)  Mitoxantrone 20 (1.4%) | **Primary Outcome:** new-  onset HF  **Secondary Outcome:**  HF exacerba-  tion, new-onset arrhythmia (defined as atrial fibrillation or  atrial flutter (AF/AFL) and/or ventricular arrhythmia), myocardial infarction, all-cause mortality, and all-cause hospitalization.  **Secondary Safety Outcome:**  serum creatinine  levels and the incidence of acute kidney injury | 2 years | Acute renal failure (22%) | SGLT2is show safety and effectiveness in  mitigating potential cardiotoxicity in patients with cancer  receiving anthracycline-based chemotherapy. The utiliza-  tion of SGLT2is is associated with a lower incidence of HF,  HF exacerbation, and arrhythmia in this patient population. |
| Perelman | 2024 | 24 patients treated with SGLT2i  **empagliflozin (83%),**  **dapa-**  **gliflozin (17%)** | 95 patients treated without SGLT2i | Israel | Diabetes  Diagnosis of cancer also with metastasis  ICIs therapy from November 2015 to August 2022  ≥18 years  SGLT2i therapy prior to ICIs therapy initiation | < 18 years | Not Specified | SGLT2i  Group: 50 ± 16  No SGLT2i  Group: 57 ± 9 | NSCLC 29 (24%)  Melanoma 19 (16%)  Renal Cell Carcinoma 27 (23%)  Hepatocellular Carcinoma 23 (19%)  Breast 6 (5%)  Cervical Squamous 6 (5%)  Other 9 (8%) | ICIs therapies  pembrolizumab (anti-  PD-1) (24%)  nivolumab (anti-PD-1) (8%)  avelumab  (anti-PD-L1) (8%)  atezolizumab (anti-PD-L1) (30%)  combined ipilimumab (anti-CTLA-4) + nivolumab  (30%) | **Primary Outcome:** all-cause mortality  **Secondary Outcome:** major adverse cardiovascular events (MACE), defined as the composite of  myocarditis, ACS, HF exacerbation (including HF hospitalizations or emergency room visits due to either denovo  or acute on chronic HF diagnosis), and arrhythmia  (including atrial fibrillation (AF), atrial flutter, ventricular  tachycardia and ventricular fibrillation). | 2.4 years | Not specified | SGLT2i therapy was associated with a lower all-cause  mortality rate in patients with cancer and DM2 treated  with ICIs therapy, in addition to lower events of AF and  Myocarditis. |
| Bhatti | 2024 | 8675 patients treated with SGLT2i | 8675 patients treated without SGLT2i | U.S.A. | Diabetes  Diagnosis of cancer  ≥18 years  From January 1, 2013, to October 31, 2022. | Prior documented history of cardiomyopathy or HF | 0 (0%) | Not Specified | Breast 4493 (26%)  Lymphomas 3496 (20%)  Multiple myeloma  and myelodysplastic syndromes 2308 (13%)  Genitourinary 789 (5%)  Gastrointestinal 3374 (19%)  Gynecologic 1241 (7%)  Respiratory and intrathoracic organs 616 (3%)  Mesothelial and soft tissue 1033 (6%)  Metastatic malignancy 2789 (16%) | Anthracyclines 4261 (25%)  Antimetabolites 3338 (19%)  Monoclonal antibodies 3597 (21%)  Small-molecule TKIs 2955 (17%)  Proteasome Inhibitors 1442 (8%)  Alkylating agents 1242 (7%)  Aromatase inhibitor 515 (3%) | **Primary outcome:** Incident CTRCD,  defined using ICD-10 codes for new onset cardiomyopathy or HF or requiring intravenous loop diuretic  agents at any time within 12 months from the index  event after excluding ischemic heart disease as an  etiology.  **Secondary endpoints:**  HF exacerbations, all-cause mortality, all-  cause hospitalization or ED visit, new onset atrial  fibrillation/ flutter, new onset metastatic cancer, and the need for further systemic antineoplastic therapy. | 1 years | Gastrointestinal Bleeding (0.68%)  Pneumonia (6.28%) | The baseline use of SGLT2is was safe  and associated with a signi cantly reduced risk of  CTRCD, HF exacerbations, all-cause mortality, all-  cause hospitalizations/ED visits, new onset atrial  brillation/ utter, new onset metastatic cancer, and  the need for systemic antineoplastic therapy. |
